# Ivermectin to reduce malaria transmission I. Pharmacokinetic and pharmacodynamic considerations regarding efficacy and safety

**DOI:** 10.1186/s12936-017-1801-4

**Published:** 2017-04-24

**Authors:** Carlos Chaccour, Felix Hammann, N. Regina Rabinovich

**Affiliations:** 1 ISGlobal, Barcelona Ctr. Int. Health Res. (CRESIB), Hospital Clínic-Universitat de Barcelona, Barcelona, Spain; 2 Centro de Investigação em Saúde de Manhiça, Maputo, Mozambique; 3 Instituto de Salud Tropical Universidad de Navarra, Pamplona, Spain; 4 Division of Clinical Pharmacology & Toxicology, University Hospital Basel, Basel, Switzerland; 5 Harvard T.H. Chan School of Public Health, Boston, USA

**Keywords:** Ivermectin, Endectocide, Pharmacokinetics, *Anopheles*

## Abstract

Ivermectin is an endectocide that has been used broadly in single dose community campaigns for the control of onchocerciasis and lymphatic filariasis for more than 30 years. There is now interest in the potential use of ivermectin regimens to reduce malaria transmission, envisaged as community-wide campaigns tailored to transmission patterns and as complement of the local vector control programme. The development of new ivermectin regimens or other novel endectocides will require integrated development of the drug in the context of traditional entomological tools and endpoints. This document examines the main pharmacokinetic and pharmacodynamic parameters of the medicine and their potential influence on its vector control efficacy and safety at population level. This information could be valuable for trial design and clinical development into regulatory and policy pathways.

**Electronic supplementary material:**

The online version of this article (doi:10.1186/s12936-017-1801-4) contains supplementary material, which is available to authorized users.

## Background

Vector control has been a fundamental pillar for the remarkable achievements in malaria control 2000–2015 [1]. Residual transmission [2, 3] and insecticide resistance [4] are some of the challenges faced for sustaining the gains of vector control. Innovation is required to reach the ambitious goals proposed by the Global Technical Strategy for Malaria 2016–2030 [5].

Ivermectin is a mixture of two semi-synthetic analogs of the fermentation products of *Sterptomyces avermitilis*. It belongs to the macrocyclic lactone complex; its chemical structure has been reviewed elsewhere [6]. Ivermectin is an anti-parasitic medicine approved for the treatment and control of human onchocerciasis, lymphatic filariasis (LF), strongyloidiasis [7] and scabies [8]. It is also an endectocide, a drug capable of killing arthropods that feed on a treated individual, including *Anopheles* mosquitoes. This property makes mass drug administration (MDA) with ivermectin a potential tool to reduce malaria transmission [9, 10]. Such an intervention has the potential to reach malaria vectors that feed on the temporal and spatial gaps left by core vector control interventions (long-lasting insecticidal nets (LLINs) and indoor residual spraying (IRS).

This paper reviews the pharmacokinetic and pharmacodynamic properties of ivermectin that can affect the efficacy and safety of MDA campaigns for malaria transmission reduction.

## Essential pharmacology

### Mechanism of action

Ivermectin blocks synaptic transmission in invertebrates by binding to glutamate-gated chlorine channels in nerve and muscle, leading to hyperpolarization, paralysis and death of the invertebrate, including mosquitoes. These channels are part of the Cys-loop family of ligand-gated ion channels and ivermectin has consequently been shown to have additional effects on other members, for instance the gamma-aminobutyric acid (GABA), histamine, and pH-sensitive chloride channels [7, 11].

In mammals, ivermectin acts as an allosteric agonist of GABA_A_ receptor, another member of the Cys-loop family of ligand-gated ion channels. These receptors are located on neurons in many central nervous system regions (incl. the cerebral cortex, the limbic system, and thalamus) and increase chloride conductance, resulting in hyperpolarization and less formation of action potentials [12]. In vertebrates, GABA is a major inhibitory transmitter. The net effect of GABA_A_ receptor stimulation is central nervous depression, which defines the syndrome of ivermectin toxicity in vertebrates.

### The glutamate-gated chlorine channel in *Anopheles gambiae*

The glutamate-gated chlorine channel (GluCl) from *Anopheles gambiae* has recently been characterized [11]. These channels are predominantly expressed in some of the organs involved in motor and sensory systems, which explains the paralytic and other effects of the drug on mosquitoes, even at sub-lethal concentrations.

In *An. gambiae*, the gene for the GluCl can be expressed in four isoforms, but only one is insensitive to ivermectin. Little is known about the expression of these channels in wild mosquito populations. Mosquito resistance to ivermectin has not been reported, but theoretically selective over-expression of the ivermectin-insensitive isoform could develop as a resistance mechanism. However, the reduced fertility seen in mosquitoes taking sub-lethal doses of the drug [13, 14] could help delay this possibility.

### Pharmacokinetics

The following discussion refers to pharmacokinetics of ivermectin in humans. Veterinary references are explicitly mentioned as such.

#### Absorption

Ivermectin is readily absorbed after oral administration. The absorption half-life ranges from 0.5 to 2.5 h [15, 16]. There are appreciable differences in systemic bioavailability (F) depending on mode of administration and disease state; ethanol based liquid formulations have up to twice the availability of solid formulations (AUC ratio 1.08–2.29) [15]. Infestations with worms such as *Strongyloides* can lead to paralytic ileus and severely impaired absorption of ivermectin. This has led to several patients requiring treatment with parenteral veterinary formulations [17, 18]. Time since last meal does not seem to influence ivermectin's bioavailability, although this is still subject to debate [19, 20].

Ivermectin is subject to presystemic metabolism and efflux in the gut. Intestinal cytochrome P_450_ 3A4 (CYP 3A4) can degrade ivermectin [21], and the active efflux pump P-glycoprotein (P-gp, MDR1, ABCB1), located luminally on intestinal enterocytes, transports absorbed ivermectin from the enterocyte back into the lumen [22]. Drugs or xenobiotics can induce or inhibit the activity of these mechanisms [23], as can pharmacogenetics differences, most notably in P-gp expression [24, 25].

As a lipophilic and comparatively heavy compound, ivermectin is thought to be subject to enterohepatic circulation (EHC) [16]. This is further supported by ivermectin being a substrate for important biliary efflux pumps (P-gp, and Breast cancer resistance protein (BCRP, ABCG2) [26]. Presence of EHC can increase the total exposure of a compound in that it can be absorbed multiple times, with high peak after initial administration and subsequent peaks after the compound has been excreted into bile and then reabsorbed again in the small intestine. At low doses, the peak concentration (Cmax) is proportional to the dose, but this proportionality is lost with doses equal or higher than 150 mcg/kg [27]. After a single oral dose of 150 mcg/kg, the peak is around 40 ng/ml [7, 15, 28]. The reported time needed to reach Cmax (Tmax) varies but is generally accepted to be approximately 4 h [28].

**Fig. 1:**
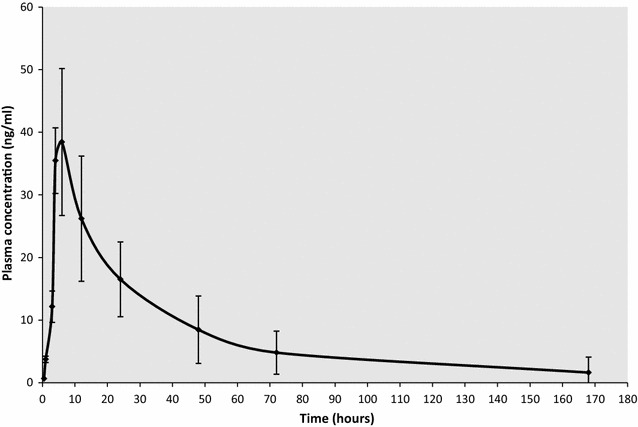
The PK curve of ivermectin. Plasma concentration of ivermectin in 10 Sudanese patients infected with onchocerciasis after a single oral 150 mcg/kg dose (Data from Elkassabi [28])

Figure 1 represents the PK curve observed by Elkassabi [28] in Sudanese patients. The relationship between plasma concentration and the mortality of mosquitoes feeding on treated individuals is reviewed in the efficacy section below.

#### Distribution

Ivermectin is highly lipophilic, shows a great degree of protein binding (>90%), and distributes widely in the body with a volume of distribution (V_d_) of 3.1–3.5 l/kg. Owing to its lipophilicity, ivermectin partitions to adipose tissue, which increases V_d_ and leads to accumulation with prolonged elimination, as drug distributes back to plasma from fatty tissue [16, 29]. This can explain the different pharmacokinetic pattern seen in women and volunteers with higher body mass index. Protein binding becomes important in populations with high prevalence of malnutrition: there, lower plasma protein levels (especially hypoalbuminemia) will result in higher free concentrations of ivermectin and, subsequently, more drug effect and toxicity.

Distribution to the brain is hindered by the blood–brain-barrier. Specifically, this is mediated by ivermectin's size, which is not conducive to passive diffusion, and the presence of efflux pumps, for which ivermectin is a substrate. The primary efflux pump is the P-gp (of which ivermectin is also an inhibitor), although BCRP can also transport ivermectin [22, 26]. The blood–brain-barrier therefore restricts ivermectin’s access to its toxicity target in mammals, the central nervous GABA_A_-receptor and forms the basis for ivermectin's good tolerability. P-gp expression at birth is quite low and reaches adult levels only after 6 months. This plays a large role in the susceptibility to central nervous effects from opioids [30] and possibly also for other P-gp substrates such as ivermectin. No controlled trials of ivermectin pharmacokinetics and safety have been performed in neonates and infants. In rats, however, ivermectin significantly increased post-natal mortality, presumably through exposure from maternal milk [31].

#### Metabolism and elimination

The plasma half-life is approximately 18 h [7]. Ivermectin is metabolized by the CYP3A4 in gut and liver [32]. The hepatic cytochrome P_450_ system at birth has 30–50% of the activity of adults [33, 34]. By consequence, failure to adjust to weight but also for decreased hepatic clearance could theoretically lead to higher than expected ivermectin exposure and toxicity in neonates and infants. Less than 1% of ivermectin is excreted unchanged in the urine (i.e. renal insufficiency will have little impact on pharmacokinetics), with most of the drug being eliminated through bile and faeces.

Ivermectin's metabolites are present at very low concentration, which makes isolation and structural characterization challenging. Authors have resorted to first identify metabolites in vitro by means of liver microsomes before attempting an in vivo characterization [27, 35]. The correlation of both systems is good in several species tested. Following this methodology, three polar metabolites: 24-hydroxymethyl-H_2_B_1a_, 24-hydroxymethyl-H_2_B_1a_-Monosaccharide and 24-hydroxymethyl-H_2_B_1b_ account for up to 50% of all metabolites in liver tissue of cattle, rat and sheep in the first 14 days after dosing [27, 35]. In swine, more than two-thirds of liver residues are composed of 3″-*O*-desmethyl-H_2_B_1a_ and 3″-*O*-desmethyl- H_2_B_1b_ [27, 35].

In humans, studies with radio-labelled ivermectin show that peak plasma concentration of metabolites is about twice that of the parent drug and occurs later, at 7 h (vs four for parent drug) [36]. Plasma metabolites are less polar than the parent drug and could be fatty acid ester conjugates of the monosaccharides or aglycone of the parent drug [36]. The major metabolites excreted are 3″-*O*-desmethyl-H_2_B_1a_ and 3″-*O*-desmethyl-H_2_B_1a_-Monosaccharide in urine and faeces respectively [36]. The plasma half-life of metabolites is about 72 h, fourfold that of the parent drug. If these metabolites have mosquitocidal activity, this could explain recent findings of a “post-ivermectin” effect in which mosquitoes feeding on treated volunteers long after the parent drug is no more identifiable in plasma still show an increased mortality [37, 38].

Ivermectin is metabolized by CYP3A4 [32] but in vitro studies suggest it does not significantly inhibit its metabolizing activity or that of CYP2D6, CYP2C9, CYP1A2, and CYP2E1, all involved in its metabolism to a lower extent [7]. There is, however, a theoretical possibility of interaction with CYP3A4 inhibitors (such as protease inhibitors) or inducers such as rifampicin.

Ivermectin is both a substrate and a potent inducer of the P-gp. P-gp plays a role in the transportation of ivermectin to the intestinal lumen and in preventing its crossing of the blood-brain barrier [39]. P-gp inhibitors (such as antifungal azoles) can increase ivermectin plasma levels in animals [40, 41]. Post-marketing reports of increased International Normalized Ratio (INR) have been rarely reported when ivermectin was co-administered with warfarin [7].

The drug–drug interactions of ivermectin with artemisinin-based combination therapy (ACT) have not been well explored. Co-administration with artemether–lumefantrine was well tolerated in a small study in Burkina Faso [42], data on its safety in combination with dihydroartemisinin-piperaquine will be available from 141 participants in the IVERMAL trial [43] and further evidence on its safety in combination with dihydroartemisinin-piperaquine and primaquine will be available from the IMSEA trial [37].

## Assessing the efficacy of ivermectin to kill mosquitoes

Efficacy is defined as the killing effect of ingestion of ivermectin by mosquitoes via blood (either through a direct-skin blood meal or through in vitro/membrane feeding methods). The evidence supporting this lethal effect has been reviewed extensively [9, 10, 44] and will not be re-visited here (see Additional file 1 for all studies). However, studies to assess the efficacy of ivermectin in reducing the survival of mosquitoes are not standardized. A typical approach is to allow a sample of vectors to feed on blood containing the drug or on a treated subject. Resulting mortality is assessed at intervals and reported in different formats.

### The concept of lethal concentration 50 (LC_50_)

The LC_50_ is a commonly seen metric of ivermectin killing effect on mosquitoes [45, 46]; it is the concentration of ivermectin in the imbibed solution or blood meal that kills 50% of the mosquitoes during a defined period of observation. It is a measure of efficacy similar to the minimum inhibitory concentration used in bacteriology and it should not be misconstrued that the goal is to kill only 50% of the feeding mosquitoes. The LC_50_ will vary according to the time point chosen for the mortality assessment. At a given drug concentration and mosquito species, the 3-day LC_50_ will be higher than the 9-day LC_50_, i.e. less drug is needed to kill 50% of the mosquitoes in 9 days, due to the addition of naturally occurring deaths. An alternative approach would be to determine the time to median mortality at any given concentration, but this has not been commonly used.

The feeding method used to determine the LC_50_ could also influence the measurement outcome. Ivermectin is highly lipophilic, it is found in higher concentrations in dermal and adipose tissue than venous plasma [29]. It is hypothesized that the resulting concentration gradient between the adipose tissue and the capillary blood may increase the drug concentration in the capillaries when compared to venous blood. This may be relevant as mosquitoes imbibe blood from subdermal capillaries and thus may ingest higher concentrations of ivermectin than would be predicted from the drug concentration in venous samples, i.e. mosquitoes feeding directly on the skin of a volunteer may have higher mortality than mosquitoes feeding on the blood of the same volunteer in a membrane-feeding device. Evidence is being generated with aim to clarify confounding of mortality measures by route of administration to mosquitoes, and perhaps other factors.

### Pharmacokinetic considerations regarding efficacy

The efficacy of ivermectin to reduce transmission is expected to be a function mainly of its lethality to the vector population. Additional benefit will be obtained from a change in the age structure of the mosquito population: in areas of ivermectin MDA, there is increasing mortality of older biting females, this skews the mosquito population towards younger (less infectious) ages and reduces transmission beyond the initially seen lethality for up to 3 weeks [10, 47]. Additionally, older mosquitoes seem to be more susceptible to ivermectin that their younger counterparts [48].

Along these lines, the sublethal effects of the drug on mosquito fertility and flying capacity [13, 14, 45] will contribute to the overall effect (see “Other effects of ivermectin” below). Both lethality and sublethal effects will be closely related to drug concentration in reached in the blood of treated individuals *and* to the time this blood concentration is sustained.

All the concepts defined here refer to the mortality of mosquitoes feeding on a single treated person.

#### The mortality of mosquitoes caused by ivermectin has a dose–response gradient and is limited by the theoretical LC_100_

**Fig. 2:**
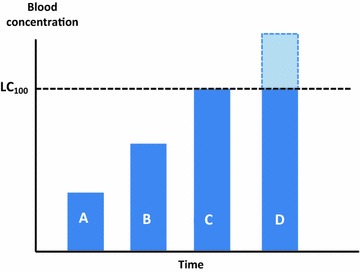
The effect of increasing concentrations of ivermectin on its efficacy. *Columns A*, *B* and *C* are expected to have increasing efficacy. The area above the LC_100_ in *column D* will not contribute to further mosquito mortality. In the absence of a long elimination tail, the efficacy of the dose of *column C* and that of *column D* will be equal. *Columns* are used for illustration. LC_100_: lethal concentration 100

The higher the concentration, the higher the mortality of mosquitoes feeding on those individuals at that time will be [42], this finding has been supported by modelling [49]. This increase in lethality will be limited by the theoretical LC_100_ (the concentration killing 100% of biting mosquitoes, this is a theoretical concentration difficult to reach in nature). Any blood concentration above this threshold will not contribute to additional mortality (one cannot kill more than 100% of the biting mosquitoes). Figure 2 illustrates this concept.

#### Ivermectin’s impact on mosquito mortality is directly related to the time there is a lethal concentration in the blood

The longer the drug remains in the blood, the more mosquitoes it will kill or disable. Any increase in duration of mosquitocidal concentrations is expected to contribute to additional mortality. Modelling shows that the time the drug remains in blood above mosquito-killing levels is the parameter that drives impact on transmission [50].

#### The lethal effect is heterogenic

**Fig. 3:**
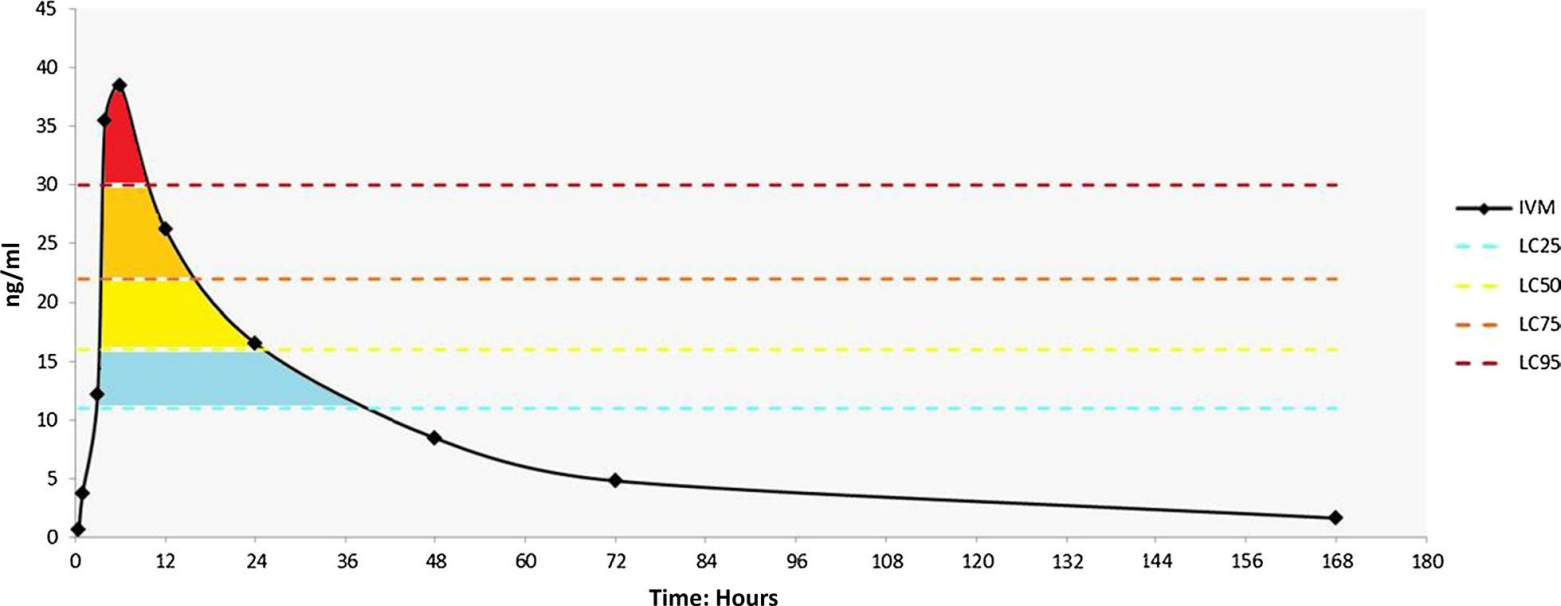
Heterogeneity of mosquito mortality according to the ivermectin plasma concentration at the time of biting. LC_50_ and LC_25_ according to Kobylinski et al. [46]. The LC_95_ and LC_75_ shown have not been determined and are shown for illustration purposes. *LC* lethal concentration

The lethality observed in any mosquito population feeding on a treated individual after a single oral dose will not be uniform. It will vary according to the plasma levels at the time of biting in close relationship with the PK of the drug. Figure 3 illustrates this concept. The total effect will be the sum of the proportions dying at different time points.

#### The lethal effect could be a function of the area under the curve

The blood concentration and the time the drug remains in the blood can be represented by the area under the curve (AUC). Because the blood concentration above the theoretical LC_100_ cannot add to lethality, the efficacy can be expected to be a function of the AUC that is below the LC_100_. The AUC below LC_100_ will vary according to the magnitude of the single dose given, the number of doses, the administration route, the absorption and distribution rates of the drug as well as its metabolism and elimination. Secondary release from adipose tissue after accumulation could also play a role.

#### Conceptually, the ideal ivermectin dose would maximize the time drug level is near the LC100 without wasting drug going beyond the level at which most mosquitoes are killed

**Fig. 4:**
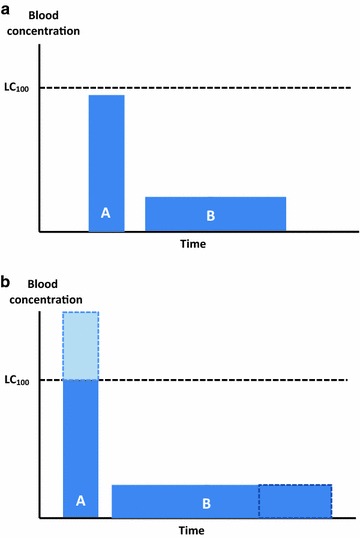
AUC of different dosing schemes and their potential relationship with efficacy. **a** The area of both *columns* is the same (*A* = *B*), hence, in the presence of constant biting rate, the total number of mosquitoes killed by A and B might me similar, even if B does not reach the same Cmax. **b** If the theoretical LC_100_ is surpassed (*light blue area*), the drug consumed to reach such plasma levels is partly wasted because it does not contribute to efficacy and may in turn increase the possibility of side effects. *Columns* are used only for illustration. *LC*_*100*_ lethal concentration 100

Given the heterogeneity of mosquito lethality in time, in the (theoretical) presence of a constant biting rate, a “peaked” curve with a Cmax close to the LC100, but with a narrow base, can have the same efficacy of a wider curve, even if the Cmax is lower. Figure 4a illustrates this concept. Ivermectin MDA could be however tailored to make the Cmax coincide with the peak biting activity of the local vectors [11].

Following this rationale and considering the point illustrated in Fig. 2, a large dose yielding a “peaked” curve with Cmax high above the LC_100_ could be less efficacious than a dosing scheme yielding the same area under the curve without surpassing the LC_100_. This is because the AUC above the LC_100_ will not directly contribute to the efficacy. This is illustrated in Fig. 4b.

#### Time above lethality target

**Fig. 5:**
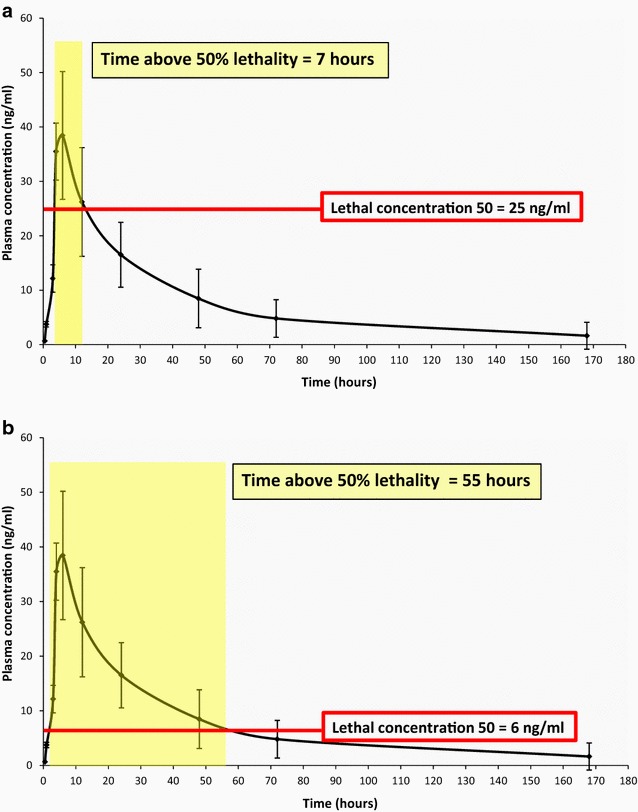
The time above lethality and the “mosquitocidal window”. An illustration of how the lethality target selected and the susceptibility of the local vectors can affect the efficacy of ivermectin to reduce malaria transmission. The *curves* represent the PK of ivermectin after a single oral 150 mcg/kg dose according to Elkassaby [28]. In both* panels*, the lethality target is the LC_50_. The vectors of **a** are less susceptible and higher concentrations are required to kill 50% of them, the time above lethality target is 7 h. The vectors of **b** are much more susceptible, this increases the time above lethality eightfold. *LC* lethal concentration

Modelling can help generate a robust hypothesis on the mosquito lethality target at a population level. This will be a function of the individual dose per body weight. The time above lethality target is related to the area under the curve but takes into consideration the susceptibility of the local mosquitoes. It can be expressed in time as a “mosquitocidal window”. Figure 5 illustrates how the susceptibility of the local vector can influence this variable.

#### Dose–response curves

**Fig. 6:**
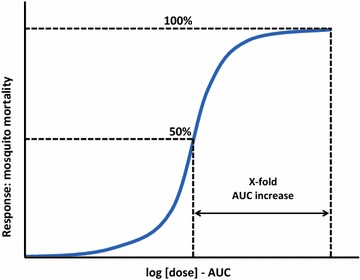
Logarithmic ivermectin dose–response curve as mosquitocidal. In the case of ivermectin, the dose will be a function of the AUC and the response a function of the total mosquito mortality plus the sublethal effects. *AUC* area under the curve

The slope of the curve will represent the logarithmic increase in the AUC below LC_100_ needed to kill a higher proportion of mosquitoes. Although recent data suggests the relationship between plasma concentration and mosquito mortality is linear at the individual level [42], at the population level the relationship AUC-efficacy is unlikely to be so. Figure 6 illustrates this concept.

### Options to increase the efficacy of ivermectin

Higher doses per body weight, multiple dose regimens, or slow-release formulations are all theoretical ways to increase the AUC and hence the efficacy. The duration of sublethal concentrations can play an important role in general efficacy as vectors imbibing sublethal concentrations can have a higher mortality rate due to impaired motility or temporary paralysis (knock-down). Epidemiological and PK modelling can be used to plan the doses and regimens to be tested in field trials, but care must be taken to reflect the potential importance of this additional effects.

#### Higher doses (increasing the Cmax)

**Fig. 7:**
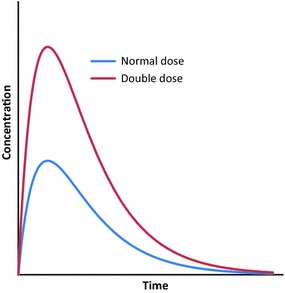
Increasing efficacy using higher doses. Using a higher dose-per-body weight in a single encounter increases the AUC by increasing the Cmax. *AUC* area under the curve

Using higher doses per body weight will result in larger AUC driven by a higher Cmax (Fig. 7). This will result in longer time above lethal concentrations because the slope of elimination will remain the same. This is the most straight-forward method because it could be implemented using the current oral formulation at a single encounter. The main challenges with this approach include the safety of a higher Cmax that could increase toxicity while and partial drug waste due to a portion of the AUC above the theoretical LC_100_. Acceptability in areas where previous lower ivermectin doses have been used must be part of integrated community engagement, also needed for the understanding of direct and indirect benefits that can be expected from this approach.

#### Multi-dose regimens

**Fig. 8:**
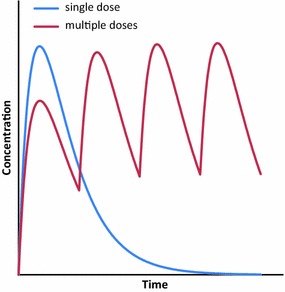
Increasing efficacy by means of multiple dosing. Using a series of multiple doses can increase the AUC while avoiding reaching plasma concentrations where the efficacy/safety ratio is lower

A multiple-dose regimen would result in a series of peak concentrations that could have cumulative effect depending on the frequency of the doses (Fig. 8). The effect of every dose would also be limited by the theoretical LC_100_ plateau. The main limitations of this approach are compliance and the logistics of multiple rounds of MDA. Additionally, the valleys caused by the intermittent dosing may result in “vulnerable windows” because levels might be in the insufficient dose range, decreasing efficacy. Preliminary data from a recent cluster-randomized trial showed a 20% reduction in clinical incidence of malaria in children under five by active case detection in areas where a 200 mcg/kg dose was given to all eligible population every 3 weeks for six doses [51].

#### Slow-release formulations

**Fig. 9:**
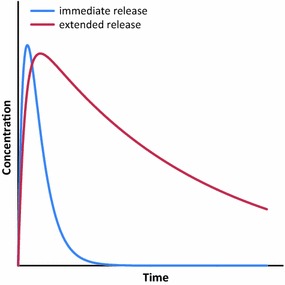
Increasing efficacy with a slow-release formulation. A long lasting formulation would increase AUC by prolonging time above lethality, without significant increase in the Cmax and theoretically improving the efficacy/safety ratio. *AUC* area under the curve

A long-lasting, slow-release formulation [52, 53] would have effect on the Cmax depending on the release rate, which, if controlled, could theoretically improve the efficacy/safety ratio (Fig. 9). The main issue with this approach is investment in R&D and the need to reassess the efficacy of the new formulation on the treatment of neglected tropical diseases.

#### Other options

One alternative to increase the AUC is to prolong the half-life of the drug (alter the elimination slope), which could theoretically be achieved with CYP3A4 and/or P-gp inhibitors. Doing so, however, may unreasonably increase the risk of toxicities and drug interactions, especially with antiretrovirals. The addition of a rather specific CYP3A4 inhibitor with no P-gp inhibition, such as voriconazole [54], would be an interesting approach as this would increase bioavailability of ivermectin while not impairing P-gp’s vital function at the blood–brain barrier.

### Other effects of ivermectin

In addition to the direct killing effect of ivermectin, there several sub-lethal effects that can increase the net impact of the drug on malaria transmission:

#### Effect on vector fertility

Several studies report diminished fertility of *Anopheles* mosquitoes after an ivermectin-loaded blood meal containing sub-lethal concentrations [13, 14]. Reduced hatching of the laid eggs has also been observed. Of note, this effect may delay but not completely avoid the appearance of ivermectin-resistant mosquitoes.

#### Effects on vector behaviour

Knock down, lesser flight performance and reduced tendency to bite have all been reported after taking sublethal ivermectin concentrations in a blood meal [45, 55]. These effects measured in the laboratory might contribute to greater mosquito mortality in the field.

#### Effects on the parasite

In the mosquito, ivermectin might inhibit plasmodium sporogony [46, 56] and could have an effect on liver schizonts as seen in vitro [57, 58] and confirmed in mouse model [57], these findings require further evaluation.

### Key knowledge gaps regarding efficacy

#### Methods

- Lack of standardized protocols for the assessment of ivermectin’s efficacy.
- Lack of correlation between the mortality observed in mosquitoes taking ivermectin through membrane vs those taking it via skin-feeding. Validating membrane feeds as a reliable, predictive assay compared to direct skin-feeding would facilitate evaluation of different approaches.

#### Lc_50_

The main gap is dearth of data on The LC_50_ determined via human direct skin-feeding. Results should be obtained for different species and strains in different sites, especially for known outdoor-biting species or main vectors of areas targeted for elimination.

#### Time above lethality

Determine the blood concentration that should be achieved with an ivermectin-based tool and how long should it be sustained in order to have a measurable impact on transmission.

#### Other effects

Assess whether ivermectin, by having a mechanism of action different from all public health insecticides available today, could help reduce the risk of insecticide resistance that is not CYP-mediated. Also the influence of ivermectin´s effects on mosquito fertility in potentially delaying the appearance of ivermectin-resistant mosquitoes.

## PK considerations regarding safety

Ivermectin has been licensed for human use for almost 30 years, and its safety has been assessed in over 70 trials. More than 2.7 billion 150–200 mcg/kg single doses have been distributed by the Mectizan Donation programme [59]. Since community use of ivermectin implies that drug will be given to at risk and infected individuals, all for indirect benefit of lowering malaria disease rates, the safety profile and the risk–benefit assessment will be critical. For use as a vector control measure, it is the combination of the blood levels and the duration of these levels that will need to have an acceptable safety profile. Both parameters are directly related to drug dosage and regimen required to reach impact. Regarding safety, the adverse effect rate is also expected to be a function of the cumulative dose. Host factors such as illnesses and co-administered drugs must also be taken into account.

### Therapeutic index

**Fig. 10:**
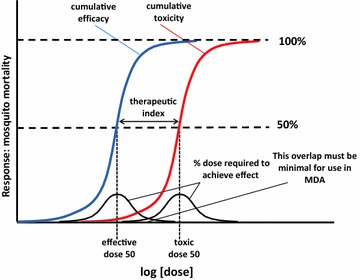
Therapeutic index. *AUC* area under the curve, *MDA* mass drug administration (Adapted from Golan et al. [61])

Drugs developed for elimination endeavours and MDA administration must have a high therapeutic index [60].The therapeutic index is a measure of the range of doses that elicit a therapeutic response without unacceptable adverse effects [61]. Using ivermectin for vector control, this relationship will be between the mosquito mortality (as a proxy for efficacy) and the AUC (as a proxy for dose per body weight and number of doses). Figure 10 illustrates this concept.

As an example, Guzzo et al. administered 1.404–2.000 mcg/kg in a single dose to 16 healthy volunteers in the US (>tenfold the usual 150-200 mcg/kg single dose for onchocerciasis) and did not report a greater adverse effect rate than in controls [62]. These findings suggest the therapeutic index of ivermectin for onchocerciasis control is at least higher than 10. For malaria control the therapeutic index will be lower as the dose needed will be higher. The findings of Guzzo et al. provide a good safety reference at 3.200 mcg/kg in a week (see Table 1). The Centers for Disease Control and Prevention recommends doses of up to 1.400 mcg/kg within a month for the treatment of severe crusted scabies [63].

**Table 1:** The safety of ivermectin at doses higher or more frequent than current approval

Reference	Population	Highest single dose	Maximum frequency	Maximum number of doses	Maximum total dose (period)	Adverse events
Awadzi et al. [100]	75 males with moderate to heavy *Onchocerca* infection	800 mcg/kg	Single	Single	800 mcg/kg (once)	No difference with controls taking the 150 mcg/kg dose
Awadzi et al. [101]	100 adult males infected with *Onchocerca*	800 mcg/kg	Days 1 and 4	2	1.600 mcg/kg (4 days)	No difference with controls taking the 150 mcg/kg dose
Guzzo et al. [62]	68 male and female healthy, adult volunteers	2.000 mcg/kg	Days 1, 4 and 7	3	3.273 mcg/kg (1 week)	No difference with controls
Kamgno et al. [67]	657 adult males infected with *Onchocerca*	800 mcg/kg	3-monthly	13	8.950 mcg/kg (3 years)	The high dose group reported a statistically higher rate of transitory mild and subjective visual side effects (No structural explanation). For all other AE comparable rates reported in all groups
Smit et al. [43]	141 adults with uncomplicated malaria	600 mcg/kg	Days 1, 2 and 3	3	1.800 mcg/kg (3 days)	Pending publication of results

The maximum total dose is the cumulative dose received by a participant during a given period which is given in brackets

*AE* adverse events

### The efficacy/safety ratio

**Fig. 11:**
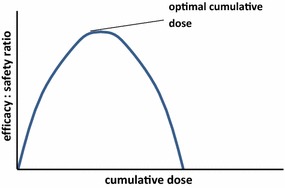
The efficacy/safety ratio. The relationship between the efficacy: adverse effect ratio and the cumulative dose

A particular AUC will elicit a specific efficacy/adverse effects ratio. This ratio is expected to increase exponentially with the AUC; in theory, once the LC_100_ has been reached, this ratio can only increase at the expense of the time above lethality. Using the therapeutic index concept described above, after a certain cumulative dose, toxicity will start to increase and the efficacy/adverse effects ratio will be reduced (Fig. 11). Increasing the dose per body weight given in a single encounter is a possible strategy to increase the efficacy. This strategy, however, must be carefully evaluated since the AUC above the LC_100_ will minimally contribute to efficacy and may in turn increase the risk of toxicity.

### Safety profile of ivermectin in community campaigns–implications for malaria

Since the creation of the Mectizan Donation Programme in 1988, more than 2.7 billion doses of ivermectin have been distributed for the control of onchocerciasis and lymphatic filariasis in Africa, Latin America, and Asia [59]. Exclusion criteria are: children under 15 kg, pregnant women, nursing mothers in the 1st week after delivery, the severely ill and those with known hypersensitivity to the drug [64]. Coverage target is normally between 65 and 80% of the whole population [65].

#### Safety of ivermectin in onchocerciasis MDA campaigns

In onchocerciasis-infected patients, adverse events (AE) to ivermectin are usually mild, transient, associated with intensity of microfilarial infection and primarily characterized as mild Mazzoti-type reactions to dying microfilaria [66]. These effects wane in subsequent administrations [67]. No significant association has been found between ivermectin plasma levels and AE recorded [68]. A recent Cochrane review of ivermectin for river blindness shows that side effects are rarely reported [69]. Outside *Loa loa* endemic areas (see below), the drug is remarkably safe.

#### Loa-associated encephalopathy

*Loa loa* is a parasitic infection that is broadly present in geographies overlapping the onchocerciasis/LF programmes. While causing limited direct disease, administration of ivermectin to individuals infested with *Loa loa* can result in encephalopathy in 0.01–0.11% of the treated population [70], if the *Loa loa* burden is high (>30.000 parasites/ml), the odds ratio can be above 1000 [70]. The syndrome includes confusion, lethargy and coma. The pathophysiology behind this syndrome is not clear, but rapid killing of *Loa* microfilariae or even defects in transmembrane efflux pumps may play a role [70, 71]. At a population level, high levels of microfilaraemia are seen in 1% of the population in areas with an overall *Loa loa* prevalence greater than 20%. This threshold was used by the Mectizan Expert Committee and the Technical Consultative Committee to define the preventive strategies recommended for ivermectin distribution in *Onchocerca* and *Loa* co-endemic areas [72]. Nonetheless, the risk of this severe adverse event excludes parts of *Loa*-endemic central Africa from ivermectin MDA campaigns for the elimination of onchocerciasis; this includes areas of Angola, Cameroon, Central African Republic, Chad, Congo, Democratic Republic of the Congo, Equatorial Guinea, Ethiopia, Gabon, Nigeria and South Sudan [73].

Several tools and strategies are emerging in response to the limitations caused by *Loa* which creates a near term window of opportunity for malaria: New diagnostic tools (loascope) allow for quantitative population screening in real time [74] and novel biomarkers could also predict burden at individual level [75]. This test and (not) treat strategy may offer a programmatic approach to addressing the *Loa* barrier to ivermectin treatment. This strategy could reduce the *Loa* burden at population level, lowering the risk of parasite-burden-related adverse reactions. Finally, single-administration of drug combos [76] can offer a rapid pathway to LF elimination, this treatment also reduces *Loa loa* burden and thus risks from ivermectin for any indication (including malaria).

#### Safety of ivermectin at doses higher or more frequent than approved for NTDs

A single ivermectin dose of 150–200 mcg/kg results in a too short-lasting mosquito-killing effect to be applicable for malaria impact. Therefore, for this indication, higher doses and/or multi-dose regimens than those currently used for onchocerciasis will be needed. A range of dosages are already recommended for different indications. The FDA-approved ivermectin dose for strongyloidiasis MDA is 150 mcg/kg (every 12 months), although the possibility of quarterly use in individual patients is also included in the label [7]. The French authorities recommend up to 400 mcg/kg for the control of lymphatic filariasis in selected areas [77]. For severe crusted scabies, up to seven 200 mcg/kg doses within a month in combination with topical treatment and keratolytics are recommended in the US [63] and Australia [78]. The possibility of using more than 3 doses for the treatment of moderate to severe crusted scabies cases is included in the Australian label [8].

Very few studies, at varying doses and frequencies, have evaluated the safety of ivermectin regimens at doses above 400 mcg/kg for control of NTDs (Table 1). Pharmacokinetic modelling suggests that a regime consisting of a daily dose of 600 mcg/kg for 3 days has the potential to sustain ivermectin concentrations lethal to *Anopheles* mosquitoes for at least 1 week [43]. This is the basis of the recently finished IVERMAL trial in Kenya [43].

The skewing of the age structure of the mosquito population for around 3 weeks after a single round of MDA for onchocerciasis (150 mcg/kg) could also support reduction in transmission of malaria. This has been used as the basis for the RIMDAMAL trial [79] which consisted on six rounds of ivermectin MDA 3 weeks apart each. Preliminary data from this cluster-randomized trial shows no significant adverse events with this those [51].

#### Safety of ivermectin during pregnancy and lactation

Pre-clinical studies in pregnant mice, rats and rabbits show teratogenicity at doses toxic to the mother (400 mcg/kg, 5.000 mcg/kg and 3.000 mcg/kg during pregnancy days 6–18 respectively) [7, 80]. Ivermectin can produce delayed development and increase pup mortality in rats at maternal doses of 1600 mcg/kg [80]. It is estimated that in *Onchocerca*-endemic areas, up to 50% of pregnant women in the first trimester are systematically inadvertently treated with ivermectin during MDA campaigns [81].

Five studies have specifically evaluated the effects of inadvertent ivermectin treatment during pregnancy (four case–control studies and one clinical trial). The results are presented in Table 2. The studies encompass a total of 839 women treated during pregnancy, including 442 women treated in the first trimester. No difference with controls is reported regarding pregnancy outcome, newborn health status or early child development. There is however no systematic database of inadvertent exposure during pregnancy to date. Based on these results, the proscription of ivermectin treatment during pregnancy was lifted for areas where women are *at high risk of blindness*. The decision to include pregnant women, however, is left at the discretion of the program directors [82].

**Table 2:** Five studies assessing the safety of ivermectin during pregnancy compared with community based controls

Reference	Country	Number of pregnant women	Number inadvertently treated	In the first trimester	Pregnancy outcome	Child mortality	Child development
Pacque et al. [81]	Liberia	939	200	171 (85%)	No difference with controls	No difference with controls	No difference with controls (follow-up 2 years)
Doumbo et al. [102]	Mali	461	82	Not stated	Data not readily assessable		
Chippaux et al. [103]	Cameroon	511	110	(93) 85%	No difference with controls	No difference with controls	No difference with controls (follow-up 1 year)
Gyapong et al. [104]	Ghana	343	50	(50) 100%	No difference with controls	No difference with controls	No difference with controls (follow-up not stated)
Ndyomugyenyi et al. [105]	Uganda	834	397^a^	All in 2nd trimester	No difference with controls	No difference with controls	Not included

^a^ Clinical trial

Low ivermectin levels are found in human breast milk after a single oral dose of 150–250 mcg/kg in healthy women with a peak at 1 h post-ingestion of 18.5 ng/ml [80, 83]. It remains detectable in human milk at very low levels (<1 ng/ml) for up to 14 days after a single dose [80]. Only nursing mothers in the 1st week after delivery are systematically excluded during MDA campaigns [64, 82].

A systematic review of the evidence regarding safety in pregnancy is needed. This is important because at population level the effectiveness of any ivermectin-based strategy will be determined by the population coverage achieved [84]. If the safety of the expected higher or more frequent doses needed for malaria is not established in pregnancy, excluding women in reproductive age is likely to reduce the efficacy of the intervention to reduce malaria transmission.

#### Safety of ivermectin in infants and children

Ivermectin is licensed for the treatment of children weighing more than 15 kg [7, 8]. In MDA campaigns a height of 90 cm is used as a proxy for 15 kg. A preclinical toxicology study in 24 neonatal (7–13 days old) rhesus monkeys showed no adverse reaction after 2 weeks of daily doses of up to 100 mcg/kg [80]. An additional study in eight immature rhesus monkeys (13–21 months old), receiving doses of up to 1.200 mcg/kg for 14–16 days showed no treatment related findings; three animals presented increased serum transaminases which was attributed to an infectious origin. In humans, there are only anecdotal case reports [85, 86] and small case series [87] of its use off-label in infants less than 15 kg.

Similarly to pregnancy, the importance of a clear guidance regarding use in children weighing less than 15 kg is directly related to ivermectin MDA efficacy. At population level, coverage will be directly proportional to efficacy [84]. Importantly, the impact of including small children on the overall efficacy of ivermectin MDA for malaria will be related to the mosquito biting rate as well as risk of transmission in this particular population.

In areas of high transmission, where most of disease burden occurs in children under 5 years, this age group is expected to receive the largest proportional benefit of ivermectin MDA to reduce malaria transmission. In the context of the RIMDAMAL trial, the main outcome measure was malaria incidence in children under 5 years of age, where most of these children did not receive ivermectin [51]. Conducting dose-ranging studies in children will allow for increased population coverage of an ivermectin-based vector control intervention.

#### Safety of ivermectin in high-risk groups

There is no renal or hepatic ivermectin dose defined [7]. Renal dose adjustments would not appear necessary for a drug for which less than 1% is excreted unchanged in urine. It is conceivable that active metabolites exist that are eliminated renally. There is little information available on the safety of ivermectin in patients over 65 years of age. A report of excess deaths (several causes) among 47 residents of a nursing home following ivermectin MDA for scabies (single dose 150–200 mcg/kg) [88] raised heated debate [89–92]. Ivermectin was licensed in Australia for the treatment of scabies in 2013 [93]. The elderly tend to have less adipose tissue and thus lower volumes of distribution for lipophilic drugs, such as ivermectin, which will result in higher plasma concentrations. They are also more prone to hypoalbuminaemia due to malnutrition, potentially yielding higher free concentrations of ivermectin. Lastly, hepatic function (and with that: capacity for detoxification) decreases with age.

There is no evident biological basis for concern on potential cardio-toxicity. Dukuly et al. [94] prospectively followed 32 men (mean age 61 years), including 20 with baseline EKG abnormalities and found no significant changes after ivermectin treatment.

HIV-infected individuals are not excluded from treatment based on their serological status [64]. Potential drug–drug interactions with anti retrovirals or TB drugs must be particularly taken into account when treating this special population (see drug interactions below).

Concerns about the theoretical risk of using ivermectin in patients with epilepsy have been resolved [82, 95].

#### Environmental concerns about ivermectin

There are three ways in which ivermectin can enter the environment: excretion from treated humans or animals, from disposal of pharmaceutical waste, or from emissions from manufacturing sites [80]. Haley et al. showed ivermectin undergoes rapid degradation in light and soil [80, 96]. This, combined with tight binding to soil and sediment prevents environmental accumulation and minimizes its potential impact on non-target organisms [80, 96]. Veterinary ivermectin formulations affect the dung fauna and there was initial concern that it may delay dung degradation [97]. More recent studies have suggest this is not the case [98].

### Key knowledge gaps regarding safety

- The safety profile of ivermectin when used at higher doses, or with longer exposure treatment schemes.
- The safety of the proposed dose/schemes in populations likely to affect coverage if excluded i.e. potentially pregnant women and children under 15 kg.
- New strategies to assess and prevent the *Loa*-related adverse effects.
- The safety of ivermectin in combination with anti-malarials and other drugs commonly used in endemic areas such as antiretrovirals, TB drugs and other antihelmintics.

## Conclusions

Ivermectin MDA has the potential to reduce malaria transmission by increasing the mortality of malaria vectors biting treated individuals, particularly those only partially affected by LLINs and IRS due to behavioural or physiological resistance. A thorough understanding of the pharmacological properties of ivermectin is pivotal to design studies aiming at providing evidence for a policy recommendation.

Ivermectin is safe in MDA campaigns at the current dose approved for onchocerciasis and LF 150–200 mcg/kg administered not more than four times a year. If used for malaria control, the dose and administration scheme will change. The efficacy of an ivermectin-based tool will be directly related to coverage; hence all population groups should be represented in the safety data collections, including women in reproductive age, children and the elderly. Additional preclinical safety studies might be needed to include these groups in clinical trials. The appropriate best time to include susceptible groups would be after the dose, formulation and administration scheme have been defined.

## Additional file

**Additional file 1.** Studies assessing the efficacy of ivermectin to kill mosquitoes taking a loaded blood meal.

## Abbreviations

AUC: area under the curve
AE: adverse events
BBB: blood–brain-barrier
BCRP: breast cancer resistance protein
GABA: gamma-aminobutyric acid
GluCl: glutamate-gated chlorine channel
HIV: human immunodeficiency virus
INR: International Normalized Ratio
IRS: indoor residual spraying
LC_50_: lethal concentration 50
LF: lymphatic filariasis
MDA: mass drug administration
NTDs: neglected tropical diseases
P-gp: p-glycoprotein
PK: pharmacokinetic
TB: tuberculosis
V_d_: volume of distribution
