# Supplementary material for: Ivermectin to reduce malaria transmission I. Pharmacokinetic and pharmacodynamic considerations regarding efficacy and safety
Source: Malar J. 2017 Apr 24;16:161. doi: 10.1186/s12936-017-1801-4 (PMC5402169; doi:10.1186/s12936-017-1801-4)
Supplement: Supplementary file 1 — Additional file 1. Studies assessing the efficacy of ivermectin to kill mosquitoes taking a loaded blood meal. [file 12936_2017_1801_MOESM1_ESM.pdf]

Reference	Method (1)	Method (2)	Species	Dose	Timespan dose to feeding	Most relevant results	Additional observations
Pampiglione 1985 [1]	treated subjects	mice	*An. stephensi*	140 to 28,000 µg/kg (once, subcutaneous)	12 hours	3-day mortality: 60% in the 280 µg/kg group<br>Controls 20% (significance not reported)	
Iakubovich 1989 [2]	membrane	in vitro mixture (blood + ivermectin)	*An. stephensi*<br>*An. sacharovi*	1 to 50 ppm	N/A	[time of mortality assessment not stated]<br>An. Stephensi LC100 1ppm<br>An. sacharovi LC100 50 ppm	
Iakubovich 1989 [2]	treated subjects	rabbits	*An. stephensi*<br>*An. Sacharovi*<br>*An. atroparvus*	340 µg/Kg (once, subcutaneous)	not available	***An. stephensi*** 4-day-mortality: 93% (significant)<br>***An. sacharovi*** No difference with control<br>***An. atroparvus*** No difference with control	
Jones 1992 [3]	membrane	blood from treated dogs	*An. quadrimaculatus*	10 to 2,500 µg/Kg (once, orally)	4 hours	2-day mortality: 92% in the 500 µg/kg group<br>Controls 3.4% (significant)	
Jones 1992 [3]	treated subjects	dogs	*An. quadrimaculatus*	10 to 2,500 µg/Kg (once, orally)	4 hours	2-day mortality: 98.6% in the 10 µg/kg group<br>Controls 4.3%	
Gardner 1993 [4]	treated subjects	dogs	*An. quadrimaculatus*	6 to 24 µg/Kg (once, orally)	4 hours	24-hour mortality: 66.9% in the 12 µg/kg group<br>Controls: 3.9% (significant)<br>24-hour LC50: between 6 and 12 ng/ml	24-hour Lethal DOSE 50: 9.9 µg/kg<br>Significant decrease in oviposition and egg-hatching from survivors
Bockarie 1999 [5]	field collections	MDA for LF	*An. punctulatus*<br>*An. koliensis*	400 µg/Kg (once, orally) + 6 mg/kg DEC	≤ 4 days	3-day mortality: 61% if collected ≤4 days post treatment<br>Controls: 1-10% (significant)<br>9-day mortality: 100% if collected ≤4 days post treatment<br>Controls: 18-23% (significant)	70% of deaths within 24 hours of collection.<br>Survival rates similar for both species
Foley 2000 [6]	treated subjects	human volunteer (1)	*Anopheles farauti*	250 µg/Kg (once, orally)	Same day to 44 days	3-day mortality: 100% if fed on the same day<br>Controls: 4% (significant)<br>3-day mortality: 80% if fed 7 days post treatment<br>9-day mortality: 100% if fed 7 days post treatment<br>Controls: 4-6% (significant)	
Fritz 2009 [7]	membrane	in vitro mixture (blood + ivermectin)	*An gambiae*<br>*An arabiensis*	0.01-1000 ppb	N/A	**Survival rates similar for both species**<br>[time of mortality assessment not stated]-LC50: 19.8 ppb<br>[time of mortality assessment not stated]-LC95: 77.7 ppb	
Fritz 2009 [7]	treated subjects	cattle	*An gambiae*<br>*An arabiensis*	600 µg/kg (once, subcutaneous)	1 to 23 days	3-day mortality: 100% if fed 1 day post treatment<br>Controls: 10% (significance not reported)<br>3-day mortality: 62% if fed 13 days post treatment<br>9-day mortality: 88% if fed 13 days post treatment<br>Controls: 10-38% (significance not reported)	No oviposition or significantly reduced oviposition from mosquitoes fed up to 17 days post-treatment
Chaccour 2010 [8]	treated subjects	human volunteers (25)	*An. gambiae*	200 µg/kg (once, orally)	1 and 14 days	3-day mortality: 84% if fed 1 day post treatment<br>Controls: 38% (significant)<br>9-day mortality: 96% if fed 1 day post treatment<br>Controls: 73% (significant)<br>No difference in mortality when fed 14 days after treatment.	A non-measured proportion of mosquitoes in the ivermectin group were noted to show lack of movement coordination, lethargy, inability to fly, and paralysis.

Reference	method (1)	method (2)	species	Dose	Timespan dose to feeding	Most relevant results	Additional observations
Kobylinski 2010 [9]	membrane	in vitro mixture (blood + ivermectin)	*An. gambiae*	0.5 to 64 ng/ml	N/A	3-day mortality: 75% in the 32 ng/ml group<br>Controls 10-20% (significance not reported)<br>3-day mortality: 90% in the 64 ng/ml group<br>Controls 10-20% (significance not reported)<br>[5 day]-LC50: 22.4 ng/ml (18-26.9)	Sublethal concentrations significantly reduced mosquito re-blood feeding rates.<br>Multiple sublethal blood meals further increased mortality.<br>Adult size of An. gambiae did not alter susceptibility to ivermectin
Sylla 2010 [10]	field collections	MDA for onchocerciasis	*An. gambiae*<br>*An. arabiensis*	150 µg/Kg (once, orally)	3 groups: pre-, 1 to 6 days and ≥ 7 days post MDA	***An. gambiae***<br>5-day mortality: 70% if collected 2 days post MDA<br>Controls: 16-22% (significant)<br>No difference with controls if collected after day 6.<br>***An. arabiensis***<br>5-day mortality: 50% if collected 1-6 days post MDA<br>Controls: <20% (significant)	
Kobylinski 2011 [11]	field collections	MDA for onchocerciasis	*An. gambiae*	150 µg/Kg (once, orally)	1-12 days post MDA		Sporozoite rate reduced by 79% in mosquitoes collected 2 weeks following MDA in treated villages vs sporozoite rate increased by 246% in control villages (significant)
Butters 2012 [12]	membrane	in vitro mixture (blood + ivermectin)	*An. gambiae*	5 different concentration (not stated)	N/A		Sublethal concentrations induced significant knockdown and inhibited recovery. No effect on the re-blood feeding rate.
Fritz 2012 [13]	membrane	in vitro mixture (blood + ivermectin)	*An. arabiensis*	0.1 to 100 ppb	N/A	9-day mortality: 70% in the 10 ppb group<br>9-day mortality: 100% in the 100 ppb group<br>Controls 10% (significance not reported)<br>[9-days]-LC50: 7.9 ppb (6.2 - 9.9)	LC95: 128.1 ppb (62.1 - 264.4)
Kobylinski 2012 [14]	membrane	in vitro mixture (blood + ivermectin)	*An. gambiae*	not stated	N/A	7-day-LC50 = 15.9 ng/ml (14.6, 17.3)	Sublethal concentrations significantly inhibited *P. falciparum* sporogony.
Bastiaens 2012 [15]	treated subjects	Swiss mice, Wistar rats and Cynomolgus monkeys	*An. stephensi*	mice and rats: 400 µg/Kg, Monkeys: 200 and 400 µg/Kg	1-5 days	Similar results for all three species.<br>3-day mortality: 70-100% if fed 1-2 days post treatment<br>Controls: 0-28% (significant)	
Naz 2013 [16]	field collections	cattle	*An. culicifacies,*<br>*An. stephensi*	200 µg/Kg (once, subcutaneous)	1 to 28 days post dose	***An. culicifacies***<br>3-day mortality: 65% if fed 1 day post treatment<br>Controls: 9%<br>9-day mortality: 80% if fed 1 day post treatment<br>Controls: 17%<br>***An. stephensi***<br>3-day mortality: 80% if fed 1 day post treatment<br>Controls: 10%<br>9-day mortality: 80% if fed 1 day post treatment<br>Controls: 25%	Significance only reported for the 12-day cumulative mortality

Reference	method (1)	method (2)	species	Dose	Timespan dose to feeding	Most relevant results	Additional observations
Yamada 2013 [17]	membrane	in vitro mixture (blood + ivermectin)	*An. arabiensis*	0.5 to 7.5 ppm (blood meal repeated daily)	N/A	24-hour mortality: 90% at 7.5 ppm<br>2-day mortality: 98% at 7.5 ppm<br>3-day mortality: 100% at 7.5 ppm	Ivermectin knocked down females almost immediately after blood feeding and killed most within 12 hours
Alout 2014 [18]	field collections	MDA for onchocerciasis or LF	*An. gambiae*	150 µg/Kg (once, orally) +/- albendazole	Pre- and 1-12 days post MDA	3-day mortality: 65% if collected 2 days post MDA<br>5-day mortality: 68% if collected 2 days post MDA<br>Controls: 16-22% (significant)<br>Survivorship reduced by 33.9% for one week post MDA	Sporozoite rates significantly reduced by >77% for two weeks following the MDAs<br>Parity rates were significantly reduced for more than two weeks after the MDAs
Kobylinski 2014 [19]	membrane	in vitro mixture (blood + ivermectin)	*An. dirus*<br>*An. minimus*<br>*An. campestris An. sawadwonrporni*	-	N/A	***An. dirus*** [7 days]-LC50 = 55.6 ng/ml<br>***An. minimus*** [7 days]-LC50 = 16.3 ng/ml<br>***An. campestris*** [7 days]-LC50 = 26.4 ng/ml<br>***An. sawadwongporni*** [7 days]-LC50 =27.1 ng/ml	Preliminary data suggests that ivermectin is sporontocidal to *Plasmodium vivax* in *An.dirus*
Ouedraogo 2015 [20]	membrane	blood from treated volunteers	*An. gambiae*,<br>*An. funestus*	200 µg/Kg (once or twice, orally)	1-7 days	***An. gambiae***<br>3-day mortality: 33% if fed 1 day post treatment (1 dose)<br>3-day mortality: 31% if fed 3 days post treat. (2 doses)<br>Controls: 6% (significant)<br>10-day-mortality: 59% if fed 1 day post treatment (1 dose)<br>10-day-motality: 66% if fed 3 days post treat. (2 doses)<br>Controls: 21% (significant)<br>10-days-LC50: 5.97 ng/ml<br>***An. funestus***<br>3-day mortality: 19% if fed 1 day post treatment (1 dose)<br>3-day mortality: 22% if fed 3 days post treat. (2 doses)<br>Controls: 3% (significant)<br>10-day-mortality: 40% if fed 1 day post treatment (1 dose)<br>10-day-mortality: 51% if fed 3 days post treat. (2 doses)<br>Controls: 5% (significant)	The artemether-lumefantrine-ivermectin combination was well-tolerated<br>4- to 7-fold increased mortality in mosquitoes feeding 1 day after ivermectin<br>Day 7 ivermectin plasma levels were positively associated with body mass index and female gender
Derua [21]	treated subjects	human volunteers	*An. gambiae*	150–200 µg/kg	24 hours	3-day mortality: 66.2%<br>Controls 4% (significant)<br>9-day mortality: 95%<br>Controls 12% (significant)	None of the An. gambiae in the ivermectin group laid eggs
Kobylinski 2015 [22]	membrane	in vitro mixture (blood + ivermectin)	*An. dirus*	not stated	N/A		Sublethal concentrations significantly inhibited *P. vivax* sporogony.
Poché 2015 [23]	treated subjects	Cattle	*An. Coluzzi*	100 & 200 µg/kg	1-21 days	**100 µg/kg**<br>3-day mortality 45-63% if fed < 7 days post treatment<br>9-day mortality 65-94% if fed < 7 days post treatment<br>(significant differences only one day post-treatment)<br>**200 µg/kg**<br>3-day mortality 53-77% if fed < 7 days post treatment<br>9-day mortality 85-100% if fed < 7 days post treatment<br>(significant differences until 7 days post-treatment)	Anopheles coluzzii carrying the kdr mutation

Reference	method (1)	method (2)	species	Dose	Timespan dose to feeding	Most relevant results	Additional observations
Seaman 2015 [24]	membrane	in vitro mixture (blood + ivermectin)	*An. gambiae*	11.75 ng/ml	N/A		Mosquito ivermectin susceptibility increased with age and previous bloodfeeding.<br>Likely midgut interactions resulting from ivermectin ingestion: blood meal digestion physiological responses, midgut microflora, and innate immune responses.<br>Gene transcription consistently affected by ivermectin ingestion.
Derua 2016 [25]	field collections	Larvae	*An. gambiae*	0.001-10 ppm	N/A	24-hour mortality: 38.4% with 0.1 ppm<br>24-hour mortality: 100% with 1 and 10 ppm	Cx. quinquefasciatus larvae approximately 10-fold more susceptible than *An. gambiae*

Studies assessing the efficacy of ivermectin to kill mosquitoes taking a loaded blood meal. Only studies based on blood meals are included, i.e. no impregnated cotton or sugary solutions. Studies using more than one feeding methodology were separated by experiment. Efforts were made to present the results in a uniform manner. 3-day and 9-day cumulative mortality were chosen as main outcomes given the duration of the gonotrophic and sporogonic cycles. If these were not available, the authors were contacted, and calculated when raw data was provided. If no response was obtained or if the data was not available, the 3-day and 9-day cumulative mortality were extrapolated from the Kaplan-Meier curves in every publication. If this was not possible, the cumulative mortality is reported together with the original time of assessment. The LC50 is reported with the time used for its assessment. Four studies do not use mortality as a primary endpoint (Kobylinski 2011 & 2015, Butters 2012 and Seaman 2015), but are included because of the importance of their results (sporozoite rate, knockdown/recovery and effect of senescence). One study on *Anopheles*/*Culex* larvae is included for potential implications of residual ivermectin in latrines.

1. Pampiglione S, Majori G, Petrangeli G, Romi R: **Avermectins, MK-933 and MK-936, for mosquito control.** *Trans R Soc Trop Med Hyg* 1985, **79:**797-799.
2. Iakubovich V, Zakharova NF, Alekseev AN, Alekseev EA: **[Evaluation of the action of ivermectin on blood-sucking mosquitoes].** *Med Parazitol (Mosk)* 1989**:**60-64.
3. Jones JW, Meisch MV, Meek CL, Bivin WS: **Lethal effects of ivermectin on Anopheles quadrimaculatus.** *J Am Mosq Control Assoc* 1992, **8:**278-280.
4. Gardner K, Meisch MV, Meek CL, Biven WS: **Effects of ivermectin in canine blood on Anopheles quadrimaculatus, Aedes albopictus and Culex salinarius.** *J Am Mosq Control Assoc* 1993, **9:**400-402.
5. Bockarie MJ, Hii JL, Alexander ND, Bockarie F, Dagoro H, Kazura JW, Alpers MP: **Mass treatment with ivermectin for filariasis control in Papua New Guinea: impact on mosquito survival.** *Med Vet Entomol* 1999, **13:**120-123.
6. Foley DH, Bryan JH, Lawrence GW: **The potential of ivermectin to control the malaria vector Anopheles farauti.** *Trans R Soc Trop Med Hyg* 2000, **94:**625-628.
7. Fritz ML, Siegert PY, Walker ED, Bayoh MN, Vulule JR, Miller JR: **Toxicity of bloodmeals from ivermectin-treated cattle to Anopheles gambiae s.l.** *Ann Trop Med Parasitol* 2009, **103:**539-547.
8. Chaccour C, Lines J, Whitty CJ: **Effect of ivermectin on Anopheles gambiae mosquitoes fed on humans: the potential of oral insecticides in malaria control.** *J Infect Dis* 2010, **202:**113-116.
9. Kobylinski KC, Deus KM, Butters MP, Hongyu T, Gray M, da Silva IM, Sylla M, Foy BD: **The effect of oral anthelmintics on the survivorship and re-feeding frequency of anthropophilic mosquito disease vectors.** *Acta Trop* 2010, **116:**119-126.
10. Sylla M, Kobylinski KC, Gray M, Chapman PL, Sarr MD, Rasgon JL, Foy BD: **Mass drug administration of ivermectin in south-eastern Senegal reduces the survivorship of wild-caught, blood fed malaria vectors.** *Malar J* 2010, **9:**365.
11. Kobylinski KC, Sylla M, Chapman PL, Sarr MD, Foy BD: **Ivermectin mass drug administration to humans disrupts malaria parasite transmission in Senegalese villages.** *Am J Trop Med Hyg* 2011, **85:**3-5.
12. Butters MP, Kobylinski KC, Deus KM, da Silva IM, Gray M, Sylla M, Foy BD: **Comparative evaluation of systemic drugs for their effects against Anopheles gambiae.** *Acta Trop* 2012, **121:**34-43.
13. Fritz ML, Walker ED, Miller JR: **Lethal and sublethal effects of avermectin/milbemycin parasiticides on the African malaria vector, Anopheles arabiensis.** *J Med Entomol* 2012, **49:**326-331.
14. Kobylinski KC, Foy BD, Richardson JH: **Ivermectin inhibits the sporogony of Plasmodium falciparum in Anopheles gambiae.** *Malar J* 2012, **11:**381.
15. Bastiaens G vGG, Hooghof J, Lindsay SW, Drakeley C, Churcher T, Verhave JP, Kocken C, Sauerwein RW, Bousema T: **Duration of the mosquitocidal effect of ivermectin.** *MW Journal* 2012, **3:**1-5.
16. Naz SM, A; Ahmad, M; Ahmad, A; Zaman, S: **Efficacy of Ivermectin for Control of Zoophilic Malaria Vectors in Pakistan.** *Pakistan J Zool* 2013, **45:** 1585-1591.

17. Yamada H, Soliban SM, Vreysen MJ, Chadee DD, Gilles JR: **Eliminating female Anopheles arabiensis by spiking blood meals with toxicants as a sex separation method in the context of the sterile insect technique.** *Parasit Vectors* 2013, **6:**197.
18. Alout H, Krajacich BJ, Meyers JI, Grubaugh ND, Brackney DE, Kobylinski KC, Diclaro JW, 2nd, Bolay FK, Fakoli LS, Diabate A, et al: **Evaluation of ivermectin mass drug administration for malaria transmission control across different West African environments.** *Malar J* 2014, **13:**417.
19. Kobylinski KCP, Alongkot; Ubalee, Ratawan; Schuster, Anthony; McCardle, Wes; Foy, Brian D; Tarning, Joel; Szumlas, Dan E; Richardson, Jason H. : **Assessing ivermectin susceptibility of Greater Mekong Subregion malaria vectors.** *Poster session presentes at: Annual meeting of the American Society of Tropical Medicine and Hygiene; 2014 Nov 2-6; New Orleans* 2014.
20. Ouedraogo AL, Bastiaens GJ, Tiono AB, Guelbeogo WM, Kobylinski KC, Ouedraogo A, Barry A, Bougouma EC, Nebie I, Ouattara MS, et al: **Efficacy and safety of the mosquitocidal drug ivermectin to prevent malaria transmission after treatment: a double-blind, randomized, clinical trial.** *Clin Infect Dis* 2015, **60:**357-365.
21. Derua YA, Kisinza WN, Simonsen PE: **Differential effect of human ivermectin treatment on blood feeding Anopheles gambiae and Culex quinquefasciatus.** *Parasit Vectors* 2015, **8:**130.
22. Kobylinski KC UR, Ponlawat A, McCardle W, Foy BD, Szumlas D, Richardson J: **Ivermectin inhibits the development of Plasmodium vivax in Anopheles dirus.** In *64th Annual meeting of the American Society of Tropical Medicine and Hygiene; 2015 Oct 25-29; Philadelphia*2015.
23. Poche RM, Burruss D, Polyakova L, Poche DM, Garlapati RB: **Treatment of livestock with systemic insecticides for control of Anopheles arabiensis in western Kenya.** *Malar J* 2015, **14:**351.
24. Seaman JA AH, Burton TA, Kuklinksi W, Foy BD, Saavedra-Rodriguez K: **The Effect of Senescence on Ivermectin Susceptibility in Malaria Transmitting Mosquitoes.** In *American Society for Microbiology General Meeting* Boston MS2014.
25. Derua YA, Malongo BB, Simonsen PE: **Effect of ivermectin on the larvae of Anopheles gambiae and Culex quinquefasciatus.** *Parasit Vectors* 2016, **9:**131.
